# Efficacy and safety of drug-eluting bead transarterial chemoembolization (DEB-TACE) plus apatinib versus DEB-TACE alone in treating huge hepatocellular carcinoma patients

**DOI:** 10.1007/s11845-021-02884-w

**Published:** 2022-01-27

**Authors:** Ningjie Li, Jiao Chen

**Affiliations:** 1 Department of Radiology, Wuhan Sixth Hospital, Affiliated Hospital of Jianghan University, Wuhan, 430015 China; 2 Department of Radiology, Edong Healthcare Group, Huangshi Central Hospital, Affiliated Hospital of Hubei Polytechnic University, No.141 Tianjin Raod, Huangshi, 435000 China

**Keywords:** Apatinib, Drug-eluting bead transarterial chemoembolization, Efficacy, Huge hepatocellular carcinoma, Safety

## Abstract

**Background:**

Apatinib, a tyrosine kinase inhibitor, inhibits angiogenesis under the tumor hypoxic environment induced by drug-eluting bead transarterial chemoembolization (DEB-TACE), which is hypothesized to have synergic effect with DEB-TACE in treating hepatocellular carcinoma (HCC) patients. This study aimed to evaluate the efficacy and safety of DEB-TACE plus apatinib in treating huge HCC patients.

**Methods:**

Totally, 73 huge HCC patients (tumor size > 10 cm) were screened and divided into DEB-TACE plus apatinib group (*N* = 34) or DEB-TACE group (*N* = 39) based on the treatment they received. Their clinical response and adverse events were retrieved. The progression-free survival (PFS) and overall survival (OS) were calculated.

**Results:**

DEB-TACE plus apatinib achieved a trend of higher objective response rate (64.7% vs. 43.6%, *P* = 0.071), but similar disease control rate (88.2% vs. 79.5%, *P* = 0.314) than DEB-TACE alone. Moreover, DEB-TACE plus apatinib reached an improved PFS (median (95%CI): 19.0 months (15.5–22.5) vs. 10.9 months (8.0–13.8), *P* = 0.025) and OS (median (95%CI): 25.1 months (20.3–29.9) vs. 13.7 months (9.8–17.6), *P* = 0.042) than DEB-TACE alone. After adjustment by multivariate Cox’s regression analyses, DEB-TACE plus apatinib (vs. DEB-TACE alone) was independently correlated with better PFS (HR: 0.420, *P* = 0.004) and OS (HR: 0.477, *P* = 0.022). Regarding safety, adverse events were mostly mild and manageable; also, they were of no difference between DEB-TACE plus apatinib and DEB-TACE alone (all *P* > 0.05).

**Conclusion:**

DEB-TACE plus apatinib achieves prolonged PFS and OS, while similar adverse events occurrence compared to DEB-TACE alone in huge HCC treatment.

**Supplementary information:**

The online version contains supplementary material available at 10.1007/s11845-021-02884-w.

## Introduction

Huge hepatocellular carcinoma (HCC), defined as tumor size above 10 cm in diameter, places a dramatic disease burden since management options are currently limited, and it usually presents with unfavorable survival [1–6]. For instance, most huge HCC patients experience tumor invasion and intrahepatic metastasis, which misses the opportunity of surgical resection as the curative treatment [2, 5, 6]. Consequently, transarterial chemoembolization (TACE) is commonly applied in treating huge HCC patients [7–9]. Despite displaying certain efficacy and safety, TACE may cause disease progression and result in an unfavorable survival profile in huge HCC patients due to neoangiogenesis arising from the hypoxic environment of tumor blood vessels [8–10]. Hence, developing novel therapies to improve the prognosis of huge HCC patients is urgent and necessary.

Apatinib, a tyrosine kinase inhibitor selectively targeting vascular endothelial growth factor receptor-2 (VEGFR-2), may synergize with TACE in treating HCC patients via inhibiting angiogenesis in the tumor hypoxic environment caused by TACE [10–13]. For instance, TACE plus apatinib achieves better short-term and long-term treatment efficacy as well as similar incidence of adverse events compared to TACE alone in treating advanced HCC patients, recurrent HCC patients, and huge HCC patients [14–18]. Drug-eluting bead TACE (DEB-TACE), as an improved and innovated type of TACE, utilizes non-absorbable microspheres as embolic agent and drug-loaded material to the target tumor region in treating HCC patients [19]. Moreover, DEB-TACE exhibits several advantages over conventional TACE (such as improved embolic effect, less safety issue, better loading and releasing profile etc.) [19]. Few studies apply DEB-TACE plus apatinib in treating huge HCC patients.

Thus, this study aimed to compare the efficacy and safety of DEB-TACE plus apatinib versus DEB-TACE alone in huge HCC patients.

## Methods

### Subjects

This study screened a total of 73 huge HCC patients who were treated by DEB-TACE or DEB-TACE plus apatinib from January 2018 to December 2020. The screening criteria were set as (i) diagnosed as HCC, (ii) largest tumor size ≥ 10 cm, (iii) at least one treatment response data was available, (iv) clinical data before operation were accessible, and (v) treated by DEB-TACE or DEB-TACE plus apatinib. The patients with other primary cancers or hematological malignancies at diagnosis were excluded from the study. After screening, 73 huge HCC patients were divided into two groups according to the different treatment regimens: DEB-TACE group, 39 patients with DEB-TACE therapy and DEB-TACE plus apatinib group, 34 patients with DEB-TACE plus apatinib therapy. The study was approved by the Institutional Review Board.

### Data collection

Patients’ characteristics were collected from medical records for the further analysis, including demographic information, disease information, tumor marker, liver function indexes, treatment history, and cycles of DEB-TACE.

### Treatment procedure

All patients in the study underwent DEB-TACE therapy, and the procedure was the same as a previous study [20]. Briefly, femoral artery was pierced by Seldinger technique, and tumor-supplying artery was identified by hepatic angiography; then the tumor-feeding artery was catheterized with microcatheters by super-selective catheterization, following that microspheres (CalliSpheres bead, HepaSpheres bead or DC bead) were applied as carriers to load pirarubicin and were slowly injected into the tumor-supplying artery. The embolization was completed when staining of tumor was disappeared. For DEB-TACE plus apatinib group, 1 week after DEB-TACE operation, apatinib was taken orally at a dose of 500 mg daily, and discontinued 1 week before the next DEB-TACE operation. After the last DEB-TACE operation, patients received apatinib continually at a dose of 500 mg daily, and the use of apatinib was continued until progression of tumor, intolerance of patients, or death of patients. Intolerance of patients was defined as patients who experienced adverse events and required dose adjustment or discontinuation when receiving apatinib. For the patients who had serious adverse events while receiving apatinib, symptomatic treatment was performed, and the dose of apatinib was halved. If there was no remission of adverse events after symptomatic treatment, apatinib was discontinued.

### Assessment

Clinical responses (including complete response (CR), partial response (PR), stable disease (SD), and progressive disease (PD)) were evaluated in accordance with the modified Response Evaluation Criteria in Solid Tumors (mRECIST) [21], and the data were obtained from the clinical records, which were assessed 6–10 weeks after initiation of first DEB-TACE operation. Then, objective response rate (ORR) and disease-control rate (DCR) were calculated furtherly. Besides, adverse events and liver function indexes (alanine aminotransferase (ALT) and aspartate aminotransferase (AST)) at 6–10 weeks after initiation of first DEB-TACE operation were retrieved for safety assessment of the treatment. In addition, follow-up data of all patients were abstracted from the medical records as well. The final date of follow-up was August 31, 2021, based on which progression-free survival (PFS) and overall survival (OS) were calculated for prognostic analysis.

### Statistical analysis

SPSS 21.0 statistical software (IBM Corp., Armonk, New York, USA) and GraphPad Prism 7.02 (GraphPad Software Inc., San Diego, California, USA) were used for analysis and plotting, respectively. Patients’ characteristics, clinical responses, and adverse events between groups were compared using Student’s *t* test, Wilcoxon rank sum test, chi-square test, and Fisher’s exact test. PFS and OS were elucidated using Kaplan–Meier (KM) curves, and the median PFS and OS with 95% confidence interval (CI) were calculated as well. The KM curves were analyzed by log-rank test and Gehan-Breslow-Wilcoxon test. Factors affecting PFS and OS were determined using multivariable Cox’s proportional hazard regression model analyses with forward stepwise (conditional) method, and all patient’s characteristics were included in the analysis. A *P* value < 0.05 indicated statistical significance.

## Results

### Huge HCC patients’ clinical features

The mean ages of patients in DEB-TACE plus apatinib group and DEB-TACE group were 58.3 ± 8.5 years and 60.4 ± 8.5 years, respectively (Table 1). Moreover, there were 6 (17.6%) females and 28 (82.4%) males in the DEB-TACE plus apatinib group then 3 (7.7%) females and 36 (92.3%) males in DEB-TACE group. Moreover, 5 (14.7%) and 2 (5.1%) patients had history of tumorectomy in DEB-TACE plus apatinib group and DEB-TACE group, respectively. By comparison, there was no difference of clinical features between DEB-TACE plus apatinib group and DEB-TACE group including demographic information, disease information, tumor marker, treatment history, and cycles of current DEB-TACE (all *P* > 0.05). The detailed clinical features of huge HCC patients are listed in Table 1.

**Table 1:** Patients’ characteristics

Items	DEB-TACE group (*N* = 39)	DEB-TACE plus apatinib group (*N* = 34)	*P* value
**Demographic information**			
Age (years), mean ± SD	60.4 ± 8.5	58.3 ± 8.5	0.304
Gender, no. (%)			0.288
Female	3 (7.7)	6 (17.6)	
Male	36 (92.3)	28 (82.4)	
History of alcohol, no. (%)	11 (28.2)	9 (26.5)	0.868
History of HBV, no. (%)	28 (71.8)	22 (64.7)	0.515
History of liver cirrhosis, no. (%)	21 (53.8)	13 (38.2)	0.182
**Disease information**			
ECOG PS score, no. (%)			0.663
0	26 (66.7)	21 (61.8)	
1	13 (33.3)	13 (38.2)	
Child–Pugh class, No. (%)			0.964
A	22 (56.4)	19 (55.9)	
B	17 (43.6)	15 (44.1)	
Largest tumor size (cm), median (IQR)	11.8 (10.5–12.7)	11.0 (10.1–13.3)	0.300
Portal vein invasion, No. (%)	11 (28.2)	14 (41.2)	0.244
Hepatic vein invasion, No. (%)	6 (15.4)	6 (17.6)	0.795
BCLC stage, No. (%)			0.129
B	23 (59.0)	14 (41.2)	
C	16 (41.0)	20 (58.8)	
**Tumor marker**			
AFP (μg/L), median (IQR)	165.4 (18.9–2556.6)	483.1 (27.8–6438.8)	0.257
**Treatment history**			
History of tumorectomy, No. (%)	2 (5.1)	5 (14.7)	0.240
History of ablation, No. (%)	4 (10.3)	4 (11.8)	1.000
History of TACE, No. (%)	10 (25.6)	13 (38.2)	0.248
**Cycles of current DEB-TACE**			0.730
1 cycle, no. (%)	23 (59.0)	18 (52.9)	
2 cycles, no. (%)	8 (20.5)	10 (29.4)	
3 cycles, no. (%)	8 (20.5)	5 (14.7)	
4 cycles, no. (%)	0 (0.0)	1 (2.9)	

*DEB-TACE* drug-eluting bead transarterial chemoembolization, *SD* standard deviation, *HBV* hepatitis B virus, *ECOG PS* Eastern Cooperative Oncology Group performance status, *IQR* interquartile range, *BCLC* Barcelona Clinic Liver Cancer, *AFP* alpha-fetoprotein, *TACE* transarterial chemoembolization

### Comparison of clinical response

In DEB-TACE plus apatinib group, there were 1 (2.9%), 21 (61.8%), 8 (23.5%), and 4 (11.8%) patients who reached CR, PR, SD, and PD, respectively, while in DEB-TACE group, 0 (0.0%), 17 (43.6%), 14 (35.9%), and 8 (20.5%) patients achieved CR, PR, SD, and PD, respectively (Table 2). By comparison, DEB-TACE plus apatinib group exhibited a trend of higher ORR (64.7% vs. 43.6%, *P* = 0.071), but similar DCR (88.2% vs. 79.5%, *P* = 0.314) as DEB-TACE group.

**Table 2:** Clinical responses

Items	DEB-TACE group (*N* = 39)	DEB-TACE plus apatinib group (*N* = 34)	*P* value
Clinical response, no. (%)			0.064
CR	0 (0.0)	1 (2.9)	
PR	17 (43.6)	21 (61.8)	
SD	14 (35.9)	8 (23.5)	
PD	8 (20.5)	4 (11.8)	
ORR (CR + PR), no. (%)	17 (43.6)	22 (64.7)	0.071
DCR (CR + PR + SD), no. (%)	31 (79.5)	30 (88.2)	0.314

*DEB-TACE* drug-eluting bead transarterial chemoembolization, *CR* complete response, *PR* partial response, *SD* stable disease, *PD* progressive disease, *ORR* objective response rate, *DCR* disease control rate

### Comparison of survival profile

**Fig. 1:**
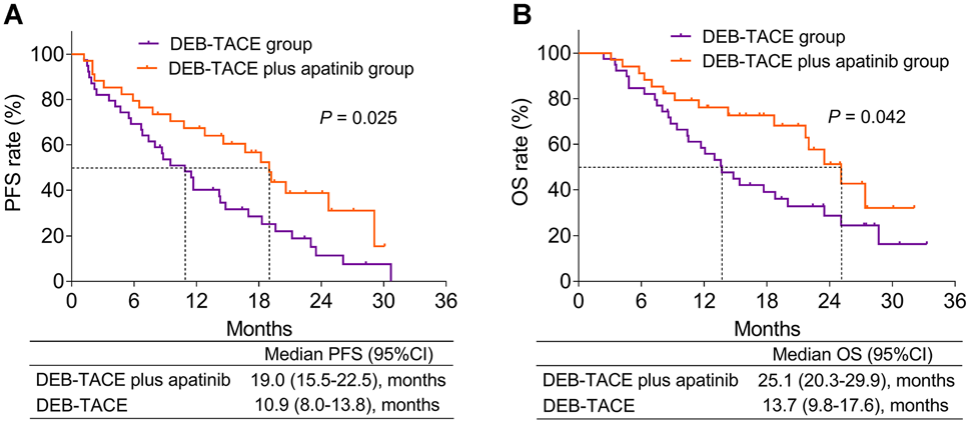
DEB-TACE plus apatinib achieved improved prognosis compared to DEB-TACE alone. Comparison of PFS (**A**) and OS (**B**) between huge HCC patients received DEB-TACE plus apatinib and DEB-TACE alone therapies

The PFS was prolonged in DEB-TACE plus apatinib group compared to DEB-TACE group (median (95%CI): 19.0 months (15.5–22.5) vs. 10.9 months (8.0–13.8)) (*P* = 0.025: Fig. 1A). Moreover, the OS was also longer in DEB-TACE plus apatinib group than DEB-TACE group (median (95%CI): 25.1 months (20.3–29.9) vs. 13.7 months (9.8–17.6)), (*P* = 0.042: Fig. 1B).

Further subgroup analysis displayed that PFS (*P* = 0.059) was similar, while OS (*P* = 0.037) was longer in DEB-TACE group plus apatinib group compared to DEB-TACE group in huge HCC patients with history of TACE (Supplementary Fig. 1A, B). Besides, PFS (*P* = 0.036) and OS (*P* = 0.049) were also prolonged in DEB-TACE plus apatinib group than DEB-TACE group in huge HCC patients without history of TACE (Supplementary Fig. 1C, D).

### Adjustment by multivariate Cox’s analyses

**Fig. 2:**
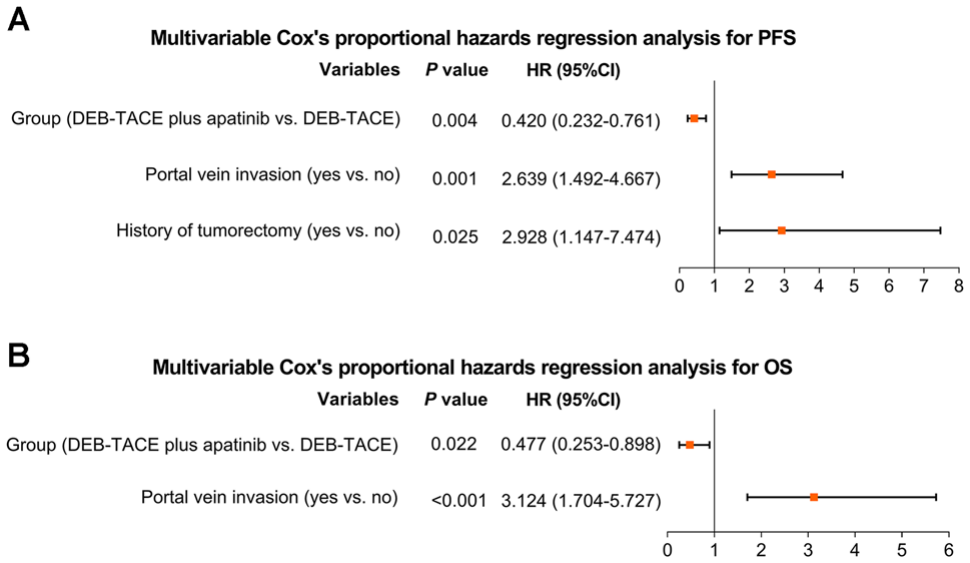
DEB-TACE plus apatinib (vs. DEB-TACE) independently correlated with prolonged PFS and OS. Multivariate Cox’s proportional hazards regression analyses for PFS (**A**) and OS (**B**) in huge HCC patients

After adjustment by multivariate Cox’s regression model, DEB-TACE plus apatinib (vs. DEB-TACE) was independently correlated with improved PFS (hazard ratio (HR): 0.420, 95%CI: 0.232–0.761, *P* = 0.004, Fig. 2A) and OS (HR: 0.477, 95%CI: 0.253–0.898, *P* = 0.022: Fig. 2B). Moreover, portal vein invasion (yes vs. no) (HR: 2.639, 95%CI: 1.492–4.667, *P* = 0.001) and history of tumorectomy (yes vs. no) (HR: 2.928, 95%CI: 1.147–7.474, *P* = 0.025) were independently associated with shorter PFS. Furthermore, portal vein invasion (yes vs. no) served as an independent factor for reduced OS (HR: 3.124, 95%CI: 1.704–5.727, *P* < 0.001).

### Comparison of adverse events

The commonly observed adverse events in DEB-TACE plus apatinib group were pain (50.0%), fever (26.5%), hand-foot-skin reaction (26.5%), nausea/vomiting (17.6%), blood pressure elevation (11.8%), diarrhea (5.9%) etc. (Table 3). Meanwhile, in DEB-TACE group, pain (46.2%), fever (17.9%), nausea/vomiting (15.4%), hand-foot-skin reaction (10.3%), diarrhea (10.3%), blood pressure elevation (5.1%) etc. were frequently reported. By comparison, these above-mentioned adverse events were of no difference between DEB-TACE plus apatinib group and DEB-TACE group (all *P* > 0.05).

**Table 3:** Adverse events

Items	DEB-TACE group (*N* = 39)	DEB-TACE plus apatinib group (*N* = 34)	*P* value
Pain, no. (%)	18 (46.2)	17 (50.0)	0.743
Fever, no. (%)	7 (17.9)	9 (26.5)	0.380
Nausea/vomiting, no. (%)	6 (15.4)	6 (17.6)	0.795
Hand-foot-skin reaction, no. (%)	4 (10.3)	9 (26.5)	0.071
Diarrhea, no. (%)	4 (10.3)	2 (5.9)	0.679
Blood pressure elevation, no. (%)	2 (5.1)	4 (11.8)	0.408

*DEB-TACE* drug-eluting bead transarterial chemoembolization

### Change of liver function indexes

**Fig. 3:**
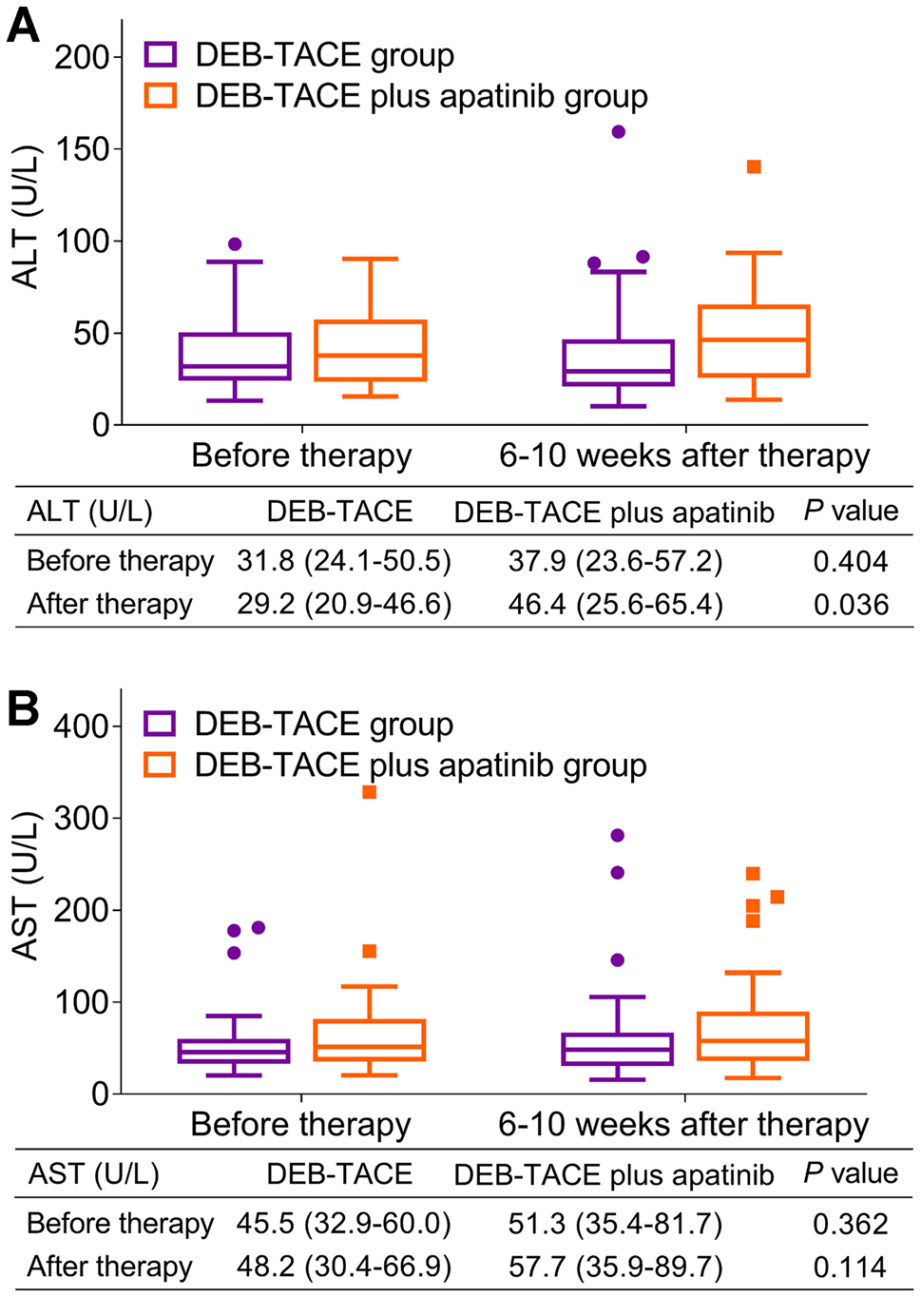
DEB-TACE plus apatinib induced a slight increase of ALT level than DEB-TACE alone. Comparison of ALT level in huge HCC patients between DEB-TACE plus apatinib group and DEB-TACE group before and after therapy (**A**). Comparison of AST level in huge HCC patients between DEB-TACE plus apatinib group and DEB-TACE group before and after therapy (**B**)

ALT level was similar before therapy (*P* = 0.404), while it was higher after therapy in DEB-TACE plus apatinib group than DEB-TACE group (*P* = 0.036) (Fig. 3A). However, there was no difference of AST level between DEB-TACE plus apatinib group and DEB-TACE group before therapy (*P* = 0.362) or after therapy (*P* = 0.114) (Fig. 3B).

## Discussion

DEB-TACE is developed as an improved technique over conventional TACE since it exhibits several advantages: (a) DEB-TACE enables a controlled manner of releasing loaded drug, (b) DEB-TACE increases intratumoral concentration and achieves a better anti-tumor effect, (c) DEB-TACE realizes an improved embolic effect by using non-soluble microspheres, (d) DEB-TACE is more convenient and feasible in the clinical practice, (e) the drug-loading speed and amount of DEB-TACE is better, and (f) DEB-TACE preciously targets HCC tumor tissue and reduces the systematic cytotoxicity [19]. However, due to enhanced tumor angiogenesis from hypoxic tumor environment caused by DEB-TACE, increased risk of disease progression and tumor recurrence in HCC patients has been reported [10, 22]. The emergence of apatinib suppresses this DEB-TACE-induced angiogenetic process arising from hypoxia tumor environment, implying its ability to synergize with DEB-TACE in HCC patients. No study compares the treatment response of DEB-TACE plus apatinib with DEB-TACE alone in huge HCC patients. In the current study, we observed that DEB-TACE plus apatinib achieved a trend of higher ORR and clinical response rate but similar DCR as DEB-TACE alone in huge HCC patients, which could be explained as that (a) the sample size of the current study was limited, leading to a low statistical power; thus, these results were not statistically significant. (b) The obvious effect of apatinib on inhibiting angiogenesis might require an accumulating period of time, which implied that 6–10-week duration was relatively short for its treatment response evaluation.

Previous study illustrates that TACE plus apatinib realizes an increased OS than TACE alone, also TACE plus apatinib (vs. TACE alone) is independently correlated with longer OS in HCC patients with macroscopic vascular invasion [14]. Moreover, several studies also exhibit that TACE plus apatinib achieves a favorable survival profile compared to TACE alone in treating advanced unresectable HCC patients, while none of them applies DEB-TACE for therapy [15–18]. Regarding DEB-TACE plus apatinib in intermediate-to-advanced HCC patients (BCLC stage B/C), DEB-TACE plus apatinib achieves a PFS of 9.5 months (95% CI: 8.1–10.9 months) and OS of 22.0 months (95% CI: 20.2–23.9 months), but it is a single-arm study which lacks a control group [23]. In the present study, we revealed that DEB-TACE plus apatinib exhibited a median PFS of 19.0 months (95% CI: 15.5–22.5 months) and median OS of 25.1 months (95%CI: 20.3–29.9 months), which were obviously favorable than DEB-TACE alone therapy in treating huge HCC patients. Also, DEB-TACE plus apatinib (vs. DEB-TACE) could independently correlate with prolonged PFS and OS, which could be explained as that (a) apatinib inhibited the angiogenesis caused by DEB-TACE, and thereby led to a synergetic effect of DEB-TACE plus apatinib in huge HCC patients [24]. Thus, DEB-TACE plus apatinib might reduce risk of disease progression and recurrence, and thereby resulted in a favorable prognosis. (b) DEB-TACE plus apatinib exhibited a numerical higher ORR than DEB-TACE alone and thus led to an improved survival profile in huge HCC patients.

Apart from the efficacy, the safety issue of TACE plus apatinib is of great attention in treating HCC patients as well. For instance, TACE plus apatinib induces a similar incidence rate of adverse events as TACE alone in advanced HCC patients (including fever, abdominal pain, nausea/vomiting, hypertension, hand-foot syndrome, diarrhea etc.) [16]. Regarding the safety of DEB-TACE plus apatinib, it induces moderate and manageable adverse events in advanced HCC patients; also, it does not influence liver function indexes [23]. In the present study, we observed that DEB-TACE plus apatinib showed a similar incidence rate of adverse events as DEB-TACE alone in huge HCC patients. ALT level was slightly elevated after therapy in huge HCC patients received DEB-TACE plus apatinib compared to those received DEB-TACE alone, which could be explained as that apatinib selectively inhibited cytochrome P450 enzyme (such as CYP2B6/2B1 and CYP2D6/2D1), which might lead to an impaired liver function and slight elevation of ALT level in huge HCC patients [25].

There were some limitations in the current study. For example, the sample size of the current study was relatively small; also, this study was a retrospective study, which might cause patient selection bias; thus, further prospective study with larger sample size to validate our findings in huge HCC patients was necessary. Moreover, apatinib-induced adverse events (such as abnormal coagulation indexes, proteinuria etc.) and the grade of adverse events were not recorded in the present study; therefore, further study could focus on this point. Furthermore, the treatment option after disease progression was not recorded since most huge HCC patients did not live in the local region requiring long-distant travelling to our hospital.

In conclusion, DEB-TACE plus apatinib achieves prolonged PFS and OS, while similar adverse event occurrence compared to DEB-TACE alone in huge HCC treatment.

## Supplementary Information

Below is the link to the electronic supplementary material.

Supplementary file1 (TIF 855 KB). Supplementary Figure 1. Subgroup analysis of survival profile in huge HCC patients. Comparison of PFS (A) and OS (B) between DEB-TACE plus apatinib and DEB-TACE alone in huge HCC patients with history of TACE. Comparison of PFS (C) and OS (D) between DEB-TACE plus apatinib and DEB-TACE alone in huge HCC patients without history of TACE.
