# Supplementary figures and images for: Efficacy and safety of drug-eluting bead transarterial chemoembolization (DEB-TACE) plus apatinib versus DEB-TACE alone in treating huge hepatocellular carcinoma patients
Source: Ir J Med Sci. 2022 Jan 27;191(6):2611–7. doi: 10.1007/s11845-021-02884-w (PMC9671984; doi:10.1007/s11845-021-02884-w)

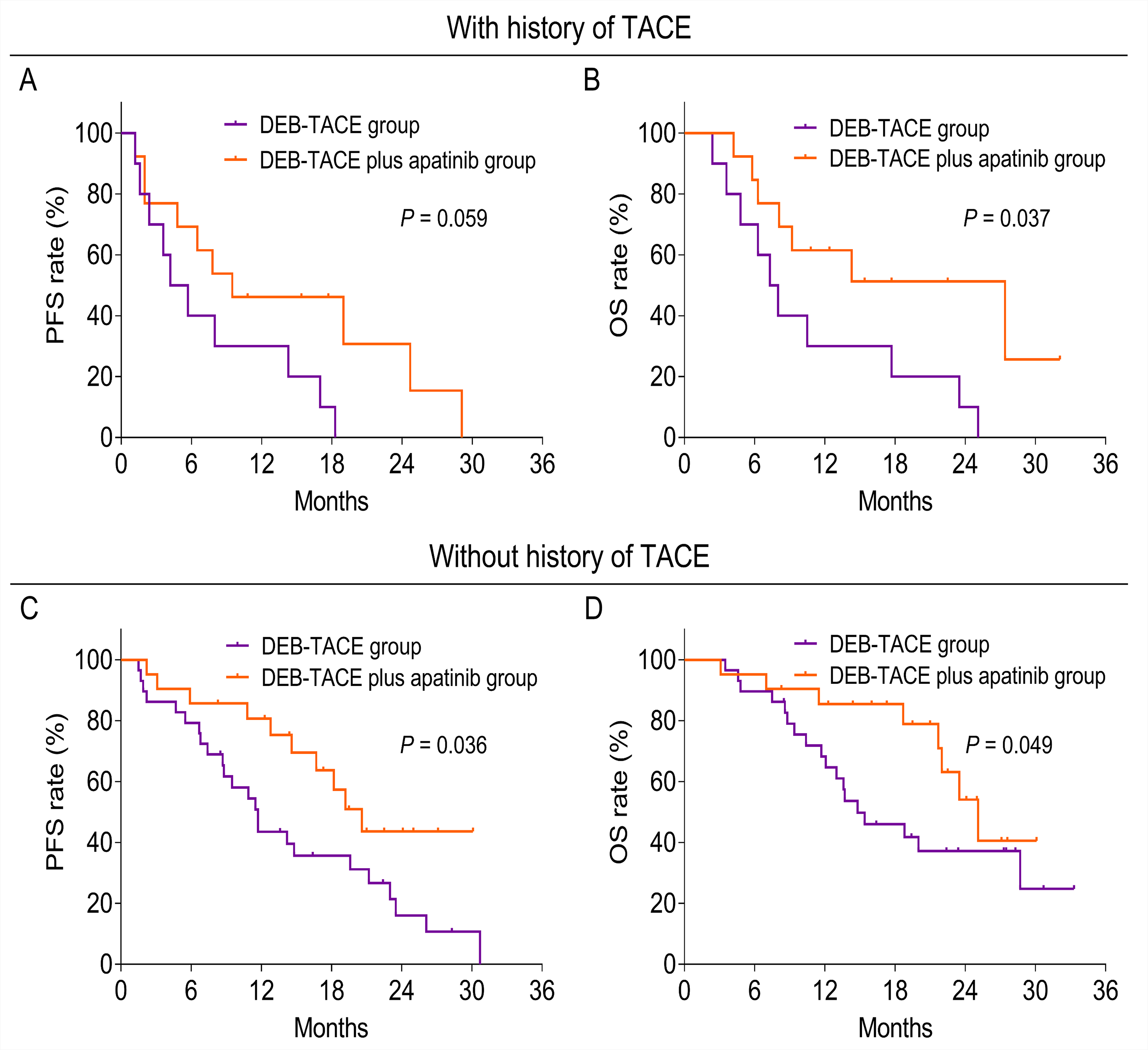

Supplement: Supplementary file 1 — Supplementary file1 (TIF 855 KB). Supplementary Figure 1. Subgroup analysis of survival profile in huge HCC patients. Comparison of PFS (A) and OS (B) between DEB-TACE plus apatinib and DEB-TACE alone in huge HCC patients with history of TACE. Comparison of PFS (C) and OS (D) between DEB-TACE plus apatinib and DEB-TACE alone in huge HCC patients without history of TACE. [file 11845_2021_2884_MOESM1_ESM.tif]
